# Internal limiting membrane peeling in macular hole surgery

**DOI:** 10.3205/000309

**Published:** 2022-06-02

**Authors:** Deepti Pradhan, Lalit Agarwal, Ichhya Joshi, Anamika Kushwaha, Kshitij Aditya, Archana Kumari

**Affiliations:** 1 Kirtipur Eye Hospital, Department of Vitreoretinal Services, Kirtipur, Nepal; 2 Biratnagar Eye Hospital, Department of Vitreoretinal Services, Biratnagar, Nepal; 3 Chandra Eye Care, Varanasi, India; 4 Prasad Eye Hospital, Ramgarh, Jharkhand, India

**Keywords:** adverse effects of ILM peeling, vital dye staining, temporal ILM flap, DONFL, Ocriplasmin

## Abstract

Since the era when macular hole was considered untreatable, macular hole surgery has come a long way to being one of the most successful surgeries. Internal limiting membrane (ILM) peeling has been an essential step of macular hole surgery since the establishment of the role of ILM in the aetiopathogenesis and progression of macular hole. However, the novel technique was not all virtuous. It had some vices which were not evident immediately. With the advent of spectral domain optical coherence tomography, short- and long-term effects of ILM peeling on macular structures were known; and with microperimetry, its effect on the function of macula could be evaluated. The technique has evolved with time from total peeling to inverted flap to just temporal peeling and temporal flap in an attempt to mitigate its adverse effects and to improve its surgical outcome. ILM abrasion technique and Ocriplasmin may eliminate the need of ILM peeling in selected cases, but they have their own limitations. We here discuss the role of ILM in the pathogenesis of macular hole, the benefits and adverse effects of ILM peeling, and the various modifications of the procedure, to then explore the alternatives.

## Abstract (de)

Seit der Ära, in der das Makulaforamen als unbehandelbar galt, hat die Makulaforamenchirurgie einen weiten Weg zurückgelegt und ist heute eine der erfolgreichsten Operationen. Das Peeling der Internal Limiting Membrane (ILM) ist ein wesentlicher Schritt der Makulaforamenchirurgie, seit die Rolle der ILM in der Ätiopathogenese und Progression des Makulaforamens bekannt ist. Jedoch brachte die neue Technik nicht nur Vorteile, sondern auch einige Nachteile, die nicht sofort ersichtlich waren. Mit dem Aufkommen der optischen Spektraldomänen-Kohärenztomographie wurden die kurz- und langfristigen Auswirkungen des ILM-Peelings auf die Makulastrukturen bekannt, und mit der Mikroperimetrie konnte die Auswirkung auf die Funktion der Makula bewertet werden. Die Technik hat sich im Laufe der Zeit vom totalen Peeling über den invertierten Flap bis hin zum nur temporären Peeling und dem temporären Flap weiterentwickelt, um die negativen Auswirkungen zu mildern und das chirurgische Ergebnis zu verbessern. Die ILM-Abrasionstechnik und Ocriplasmin können in bestimmten Fällen ein ILM-Peeling überflüssig machen, sie haben aber ihre eigenen Einschränkungen. Wir diskutieren hier die Rolle der ILM in der Pathogenese des Makulaforamens, die Vorteile und unerwünschten Wirkungen des ILM-Peelings sowie die verschiedenen Modifikationen des Verfahrens, um anschließend die Alternativen zu untersuchen.

## 1 Introduction

Macular hole (MH) is a full thickness defect in the fovea. Various theories have been proposed regarding its genesis since it was first described by Herman Knapp in 1869 [1]. The cystic degeneration theory stated that intraretinal cystic changes occur, which coalesce to form a full-thickness MH [2]. Aging and other changes in retinal vasculature were believed to cause cystic degeneration leading to the formation of a MH [3]. The role of anteroposterior vitreous traction force has long been suspected – and later supported by biomicroscopic examinations – to play an important role in the genesis of a MH [4], [5], [6]. In 1988, Gass postulated that contraction of the premacular vitreous cortex and tangential vitreous traction cause detachment of central photoreceptors and subsequent MH [7]. The events transpiring at the vitreoretinal interface before the formation of MH or during early stages of MH were better known with the dawn of the era of optical coherence tomography (OCT) when it was possible to visualize partially detached posterior vitreous with persistent attachment at the disc, the vascular arcades and the foveal center [8], [9].

The internal limiting membrane (ILM) is made of internal expansion of Müller cell footplates and a basement membrane which is composed of collagen fibers, glycosaminoglycans, laminin and fibronectin connected to peripheral fibers of the cortical vitreous [10]. ILM is thick over the macula and thin at the peripheral retina. The basement membrane layer of ILM is thin over the fovea, the optic nerve head and the major vascular arcades, where vitreous fibers are directly and strongly attached to the retina. The Müller stratum thickens into a conical shape at the foveal center connecting ILM to the external limiting membrane, which is called the Müller cap. With the liquefaction of vitreous, a precortical pocket of liquified vitreous is formed. This gradually seeps through the optic disc separating cortical vitreous from the posterior pole. However, in the eyes predisposed to MH, there is anomalous vitreofoveal adhesion which causes anteroposterior traction leading to avulsion of the Müller cap. Further expansion of the hole occurs due to tangential traction, caused by shortening of the edges of ILM and proliferation of glial and Müller cells over the ILM [11].

The staging of MH depicting its evolution was first proposed by Gass in 1988 on the basis of biomicroscopic examination. Stage 1a was foveolar detachment, which on clinical examination appeared as decreased or loss of foveal depression and appearance of yellow spot of 100–200 µ diameter due to the presence of xanthophylls in the receptors. Stage 1b was marked by foveal detachment with centrifugal displacement of the foveolar retina, bridged on top by contracted prefoveolar cortical vitreous, resulting in appearance of a yellow ring of 200–350 µ in diameter. When this progressed to full thickness macular hole of ≤400 µ central or eccentric, it was termed as stage 2 MH. As the hole enlarged further, accompanied by vitreofoveal separation with or without formation of a prefoveolar opacity, it qualified as stage 3 MH. Stage 4 was reached when vitreopapillary separation occurred, forming a Weiss ring [7], [12].

The international study group has classified vitreoretinal interface diseases into three groups: vitreomacular adhesion (VMA), vitreomacular traction (VMT) and full thickness MH. VMA does not cause any change in the foveal anatomy and hence does not interfere with vision. VMT causes distortion of the foveal contour with intraretinal changes. Both VMA and VMT are divided further, on the basis of dimension of attachment, into focal (<1500 µ) or broad (>1500 µ). MH is categorized on the basis of size as small (<250 µ), medium (250–400 µ) and large (>400 µ); on the basis of presence or absence of VMT and on the basis of etiology as primary or secondary [13].

Macular microhole (MMH) is a full thickness defect of <150 µ size at the fovea; and a partial thickness defect of the same size in the outer retina is a foveal red spot (FRS). Both are caused by vitreofoveal traction (VFT), associated either with evolution of posterior vitreous detachment (PVD) or with trauma. Patients complain of central scotoma or diminution of vision of sudden onset. FRS may also result from closure of MH or MMH. Both of them have a better prognosis than stage I and stage II MH as they heal spontaneously with the release of VFT, while 40% of stage 1 and >75% of stage 2 holes progress to further stages [14].

## 2 The inception of the MH surgery

MH was considered an inoperable condition and patients were counseled accordingly. It was Kelly who first had the idea of flattening the edges of MH with vitrectomy and gas, which he thought might improve the vision. In September 1985, he first performed the surgery but was unsuccessful as he was unaware of posterior cortical vitreous, which when later detached caused bullous retinal detachment [15].

Kelly together with Wendel continued doing more surgeries and learnt techniques to identify residual cortical vitreous. The silicon tip of the extrusion needle, when swept across the retina under active aspiration, flexed if it engaged the vitreous. This was termed “fish strike sign”. Kelly and Wendel also noticed that on proceeding with fluid air exchange, residual vitreous if present appeared as viscous substance on the retinal surface towards the end of fluid aspiration. They published their first report on MH surgery in 1991 and described a 5-step procedure: vitrectomy, removal of cortical vitreous, stripping of the epiretinal membrane (ERM) if any, complete fluid-gas (SF_6_) exchange and one week of strict prone positioning [16].

## 3 Surgical methods

Kelly and Wendel described the membrane as typically friable and difficult to remove, which in retrospect was believed to be ILM. They used a barbed 20- or 22-gauge needle, tissue forceps and a tapered extrusion needle to remove them [16]. Eckardt et al. in 1997 described ILM peeling (ILMP) as a technique to improve surgical outcome of MH [17]. They used specially designed forceps to cause “rhexis” of ILM of about 3–4 disc diameter size. With the introduction of vital dyes to stain ILM, the visualization of ILM has improved greatly. A barbed MVR blade or membrane micropick can be used to cause a linear scratch over ILM. Michels membrane pick is then used to lift ILM by horizontal, lamellar dissection, later to be removed by forceps [15]. Another method is the use of the end of a gripping forceps to pinch and tear a flap of ILM, which is then grasped and peeled off in a circular fashion, creating “maculorrhexis” [18]. A technique described by Morris and Witherspoon involves injecting Healon beneath ILM, ballooning it up, which makes it easier for the surgeon to grab and peel it [19].

The introduction of the concept of chromovitrectomy facilitated the procedure of ILM peeling as it helped in the visualization of otherwise transparent ILM. Indocyanine green (ICG) was the first dye used to stain ILM, but there were many contradicting reports, some citing a better outcome with its use, and some reporting about its side effects and worse outcomes. Several other options were then sought out, like infracyanine green (IfCG), Triamcinolone acetate (TA), trypan blue (TB), patent blue and brilliant blue (BB). ICG, IfCG and BB stain ILM better, while TB and PB are preferred for ERM. TA is the best stain for the visualization of vitreous. Among these, BB is considered the best and the safest stain for ILM [20], [21].

There has been a debate about the appropriate extent of the ILMP. Bae et al. studied the anatomical and visual outcome when the ILM was peeled with the radius of 0.75 disc diameter or 1.5 disc diameter. They concluded that a larger extent of ILMP during MH surgery results in a better outcome, so far as the improvement in metamorphopsia and alleviating the asymmetrical elongation of the foveal tissue are concerned [22]. Goker et al. suggested that the ILM should be peeled as close to the vascular arcades as possible, as they have shown in their study that a larger area of ILM peeling results in a better anatomical outcome [23]. A larger area of ILM peeling (4DD) results in a better outcome; nevertheless, in cases with a macular hole closure index >0.5, peeling of 2DD is enough to reach a comparable outcome [24].

Although ILMP is accepted as a standard procedure for MH surgery, it has seen several modifications with the aim to achieve a better outcome in more challenging cases, such as long-standing large MH and highly myopic eyes. The inverted ILM flap technique was introduced by Michalewska et al. for large MH, in which ILM is not peeled completely and is left attached at the margins of the MH. This is then inverted over the MH. A closure rate of 98% was achieved with this method [25]. Further studies and observations showed that higher closure rates and improved visual outcomes could be achieved in refractory, traumatic and highly myopic MH [26]. The mechanism of hole closure by this method is not clear, but it has been known that ILM placed over the hole contains Müller cell fragments which can induce gliosis. Kase et al. suggested that glial cells placed on the hole may produce intermediate filaments and provoke tissue remodeling within the MH [27].

Shin et al. modified the inverted ILM flap technique such that single-layered ILM covered the MH, providing more physiological and regular structure for glial cells to proliferate. They fashioned a superior ILM flap of one disc diameter, peeled the rest of the ILM around the MH, reflected the flap onto the MH, and injected perfluoro-n-octane (PFO) to keep the flap in place during fluid air exchange. With this technique, one day of post-operative face-down positioning and air tamponade were sufficient for hole closure with a more normal configuration of the fovea without folded membrane and a shorter visual recovery time [28], [29]. Viscoat has been used as an adhesive and ballast to stabilize the ILM flap during fluid air exchange and to minimize the toxic effect of ICG to retinal pigment epithelium (RPE) in large MH in highly myopic eyes [30]. Similarly, autologous blood clot was used to stabilize and seal the ILM flap [31]. Chen described a technique of large superior semicircular inverted flap with improved outcome without the need of any stabilizing substance nor of prolonged face-down positioning in MH with high myopia [32]. A novel technique of donut-shaped ILM peeling in stage 2 MH, preserving central 400 μ, thus preventing inner retinal damage, better restoration of foveal structure, and better visual outcome was described by Ho et al. [33]. Autologous transplantations of free flaps of ILM to cover holes in refractory MH have been shown to improve anatomic and visual outcomes [34]. Yet another novel technique has been described for large, chronic, full-thickness MH, in which multiple ILM flaps were inverted over each other and the hole-like cabbage leaves [35]. Chen and Yang used an anterior or posterior capsular flap to plug refractory MH [36], while Grewal and Mahmoud introduced the use of an autologous neurosensory retina (NSR) flap as scaffold and plug for refractory myopic MH [37]. For persistent, chronic and large MH following PPV and ILMP, a silicon soft tip extrusion cannula was used to actively reflux water into the MH, causing “hydrodissection” of the MH edges from RPE adhesions if any. This moved the edges further apart, which were then brought closer by brushing them with a soft-tip cannula under passive extrusion. 87.2% of them had a complete anatomical closure and all of them had type 1 closure [38]. Mohammed et al. punctured NSR at 3 sites: 2DD above, below, and at the temporal edge of the MH, and injected balanced salt solution (BSS) using a 41G subretinal cannula to cause macular detachment [39]. They used DDMS to massage detached NSR towards the center, avoiding the papillomacular bundle, and finally pinched the temporal edges with end-gripping forceps so that the edges were stretched and came closer. They reported 4 cases of recurrent MH treated with this technique and achieved type 1 closure in all of them [39].

## 4 Surgical outcome

Kelly and Wendel [16] reported that anatomical success was achieved in 58% of the cases, and improvement in visual acuity by two or more lines was seen in 73% of them. Since then, many papers have been published on MH surgery.

In two large randomized controlled trials (RCTs) [40], [41], closure was achieved in 69% and 80.6% respectively after vitrectomy, and in 4% and 11.5% following observation. Vision was significantly better after vitrectomy. The former study [40] included stage 3 and 4 MH, while the latter [41] studied stage 2 to 4 MH. ILMP, however, was not done in either of these studies [40], [41].

Eckardt et al. [17], employing ILMP in their surgical technique, demonstrated complete closure in 36 of 39 eyes (92%) and visual improvement of two lines in 77% of them. In a large retrospective comparative clinical study [42], 100% closure was seen in MH of less than 6 months’ duration treated with ILMP versus 82% in eyes treated with vitrectomy alone. Furthermore, reopening of the MH occurred in 25% of the cases in the latter group. In another group of patients with MH of more than 6 months’ duration treated with ILMP, MH closed in 97% and vision improved by 2 or more Snellen lines in 65% [42]. A prospective case series [43] reported successful closure of MH in 96% of the cases, and improvement in vision by at least 2 lines in 85% and by 3 lines in 76%. A clinical study [44] compared anatomical and visual outcome between two groups of cases following ILMP versus without ILMP. The result was in favor of ILMP with 90% anatomical closure versus only 50% in cases treated without ILMP, and significant visual acuity improvement in 62% and 44%, respectively [44]. Another study also supported this result [45], in which 97 patients in the vitrectomy-only group were compared with 79 patients in the PPV and ILMP with or without ICG staining group. A closure rate of 77.3% and 97%, and a visual gain in 65% and 77.3% was seen in the two groups respectively [45]. In a prospective non-randomized case series, Haritoglou et al. [46] followed up patients for at least 12 months. 99 eyes with MH were taken up for PPV with ILMP without the use of any dye. The primary closure rate was 87%. Functional improvement was seen in 97% with a median BCVA of 20/40 and a median gain of 5 lines [46].

In an RCT conducted in the Chinese population [47], a comparison was made between ICG-assisted ILMP performed in 26 eyes, and vitrectomy-only in 25 eyes. ILMP was found to be superior with an anatomical success rate of 92.3% and improvement in BCVA by 2 or more lines in 84.6%, in contrast to 32% of macular hole closure and 36% of gain in BCVA in the non-ILMP group. The improvement of BCVA in the ILMP group (3.7 lines) was significantly higher than that in the other group (1.5 lines) [47].

Christensen et al. [48] conducted an RCT in which 78 eyes of 75 patients with stage 2 and 3 MH without epiretinal fibrosis of ≤12 months duration were randomized into 3 groups: as 25 in vitrectomy only, 34 in vitrectomy with 0.05% ICG-assisted ILMP, and 18 in the 0.15% trypan blue (TB)-assisted ILMP group. Primary closure occurred in 55% of the eyes with stage 2 holes and in 36% of the eyes with stage 3 holes in the vitrectomy-only group, while the rate soared up as high as to 100% in stage 2 holes and 89–91% in stage 3 holes in the ILMP group. The closure rate was not significantly different between the ICG and TB group (91% vs. 89%). The functional outcome in eyes with primary closure was not significantly different among the 3 groups [48].

Tadayoni [49], in a multicentric RCT, compared ILMP with no ILMP in MH larger than 400 microns. 39 patients were in the TB-assisted ILMP group, while 41 were in the vitrectomy-only group. The closure rate was found to be significantly higher in the former group (94.9% vs. 73.2%) [49].

A large RCT conducted by the FILMS (Full-Thickness Macular Hole and Internal Limiting Membrane Peeling Study) Group [50] compared the effects of peeling and conventional surgery (vitrectomy only) on the rate of primary closure, visual acuity, quality of life, and expenses. Eyes with stage 2 and 3 full thickness MH of ≤18 months duration were included. Though the rate of primary hole closure was significantly high in the ILMP group (84% vs. 48%), the difference in distance, near visual acuity, and quality of life between them was not remarkable. ILMP was found to be cost-effective owing to fewer reoperations required [50].

## 5 Healing of macular hole

The improvement in post-operative visual acuity is occasionally not found to be significant despite anatomic closure of the MH. Evaluation with OCT in these cases has shown the presence of irregularities in the photoreceptor layer. Serial OCT during follow-up after MH surgery has enhanced our understanding of the healing process of the outer retina. Initially, proliferating glial cells filled the entire defect, which was seen as hyper-reflective foveal lesion on OCT. This was followed by restoration of integrity of ELM with migration of glial cells to the inner retina. Glial cell elimination was thought to help in the recovery cell bodies in the outer nuclear layer (ONL), which lead to reconstruction of the ellipsoid zone (EZ) or the inner segment/outer segment (IS/OS), followed by recovery of the cone interdigitation zone (CIZ) or the cone outer segment tips (COST), while glial cells disappeared completely [51], [52]. Absence of disrupted ELM in the presence of intact IS/OS junction has led to the assumption that restoration of ELM is critical for the healing of the photoreceptor microstructure [53]. Earlier recovery of ELM and glial cell elimination favored complete restoration of photoreceptors, which in turn correlated positively with postoperative BCVA [51]. Foveal cyst or outer retinal defect develop during the process of healing and disappear in about 45% of the cases with the recovery of the IS/OS line [52]. This has also been described as foveal lucency, which was shown to appear in 26% of the cases and disappear between 3 and 11 months after surgery, resulting in improvement of visual acuity [54].

Multiple healing patterns of MH have been described based on the OCT scan. Tornambe [55] has defined the MH surgery outcome as ‘elevated open’ if the edges are visible and elevated due to fluid underneath, as ‘flat open’ if the hole is visible but is in contact with RPE, and as ‘flat closed’ if the edges of the hole are not visible and the NSR is flat against RPE [55]. Imai et al. [56] described three patterns of repaired MH appearance: U type with normal foveal contour, V type with steep fovea, and W type with foveal defect of NSR. Post-operative visual acuity was the best in the U type and the worst in the W type [56]. Kang et al. categorized MH closure into two types: type 1 if MH is closed without foveal defect, and type 2 if foveal defect persists with flattening of the rim [57]. Rossi et al. [58] have proposed new MH closure patterns based on SDOCT. Type 0 is for open MH with 0A, 0B, and 0C for open MH with flat, elevated, and oedematous margins respectively. Type 1 is for closed MH, which is further classified into 1A if all the layers are reconstituted, 1B if the external layers are interrupted, and 1C if the internal layers are interrupted. Type 2 includes MH closed with autologous or heterologous filling tissues. It is classified into 2A if the filling tissue extends through all the layers, 2B if there is recovery of normal inner layers, 2C if normal outer layers are reconstituted, and 2D if there is H-shaped bridging of the filling tissues [58].

## 6 Adverse effects

With the increasing popularity and acceptance of ILMP as an integral part of MH surgery, there has been growing concern regarding its long-term effects on the structure, and thereby on the function of the retina. There have been several reports of various complications, some attributed to the dye used, especially indocyanine green (ICG), and others to the act of peeling.

### 6.1 Chormophore- or dye-related toxicity

Dye enhances the visibility of ILM, which being a transparent structure would otherwise be difficult to visualize, resulting in increased intraoperative time with the risk of light toxicity and retinal trauma. ICG was the first dye to be used after being introduced in 2000. There have been several reports of RPE changes, visual field defects, and optic atrophy attributed to its use. Clinical studies have shown controversial reports, with some claiming ICG-assisted ILMP to result in better outcome [59], [60], [61], [62], [63], while some quote adverse effects and worse functional outcome related to its use [63], [64], [65], [66], [67]. Several in-vitro and in-vivo studies have shown ICG to be toxic to RPE [68], [69], [70], [71], [72], [73] and ganglion cells/neuroretinal layer [74] in dose-dependent fashion, and its effect is seen to be augmented by illumination. Furthermore, ICG persists on the macula and optic nerve head for several months as shown by some studies [75], [76], [77], [78]. Haritoglou et al. [79] and Gandorfer et al. [80], in their ultra-structural evaluation of ICG-assisted (0.05% and 0.5%, respectively) peeled ILM, found plasma membrane of Müller cells and other undetermined retinal structures, leading them to believe that ICG causes alteration of the plane of cleavage to deeper layers of the inner retina beyond ILM [79], [80]. Da Mata et al. [81] on the other hand found the peeled structure to be only ILM when stained with 0.5% ICG. The toxicity further depends on multiple variables like concentration, volume, and commercial form of ICG used, solution used to dilute the dye, injection under air or water, duration of ICG incubation, type and duration of illumination, size of IMH, and the amount of residual vitreous [82]. To improve the safety profile of ICG, Grisanti et al. [83] have recommended the use of isoosmolar ICG solutions (osmolarity ≥290 mosm/kg) with a concentration of ≤1 mg/ml, an incubation time of 1 minute, and thorough removal of the dye, followed by exposure to illumination for ≤5 minutes [83]. The concentration advocated to be used has been further reduced to ≤0.5% or even ≤0.05% with use of 5% glucose as diluting agent to achieve isoosmolar solution [11].

Infracyanine (IfCG) being devoid of iodine and isoosmolar is presumed to be safer, and this is supported by an in-vitro study in which no irreversible damage occurred to RPE and Müller cells [84]. This was further substantiated by an animal study in which retinal injury induced by subretinal injection of 0.5% IfCG was much less significant than that by 0.05% ICG [85]. On the other hand, some studies have shown that retinal toxicity rendered by both the dyes is of similar scale [86], [87].

Trypan blue (TB) is used to stain the anterior capsule during cataract surgery and is known to be safe. With the reports of ICG toxicity on the rise, there was an inclination towards the search for other options. TB was found to stain the epiretinal membrane, but also, in a more subtle manner, the ILM. In-vitro studies have shown variable results at a higher dose, however, it is found to be non-toxic to RPE and NSR at a lower dose with and without light [88], [89]. Kodjikian et al. [90] showed that TB had detrimental effects on cultured human RPE cells when exposed for 6 days, but had no such effect on acute exposure (5 mins) irrespective of doses. On the contrary, no adverse effect was seen on RPE with acute (3 mins) or chronic (72 hrs) exposure of TB at any dose in other studies [91], [92]. However, time- and dose-dependent neurotoxicity with the use of TB in the laboratory has been reported [93]. The concentration of 0.02% to 0.06% is considered safe [94]. Clinical studies have been favorable towards TB in comparison to ICG, with better functional outcome and fewer adverse effects [95], [96], [97], while some have reported a similar outcome for both the dyes [48], [98]. One issue with the application of TB is the need of air fluid exchange for it to stain the ILM. To avoid this and to make reapplication feasible, a heavier form was formulated by mixing it with 10% glucose in a 1:1 ratio, which was found to be equally safe [99].

Brilliant blue G (BBG) was introduced by Hisatomi et al. [100] and Enaida et al. [101] as a dye capable of staining capsule and ILM as brilliantly as ICG, but with much less propensity for cytotoxicity as well as phototoxicity, and with an advantage of being user-friendly. In a study comparing BBG 0.05%, TB 0.15%, and ICG 0.5%, BBG was declared superior in terms of ease of use, was comparable to TB in surgical outcomes, and almost similar to ICG as far as staining property was concerned [97]. A meta-analysis has concluded that even though the MH closure rate is comparable, functional outcome is superior with the use of BBG than with other dyes [102]. Another meta-analysis using ranked probability results has found that the probability of achieving MH closure is significantly higher with the use of BBG than with TA, TB, ICG, or without dye [103].

Triamcinolone acetate (TA) is best known for staining vitreous in vitreoretinal surgery. Since its particles settle on the ILM, it can thereby help in identification and peeling of the ILM. According to a meta-analysis comparing outcomes of 4 different dyes and no dye, the highest probability of attaining success in terms of visual outcome was with the use of TA, and following in decreasing order were BBG, TB, no dye, and ICG [103]. A concern with the use of TA in MH surgery is deposition of its particles within the hole, which was thought to hinder its healing process or cause re-opening [104]. However, some have observed no interference of such deposits on anatomical and functional success [105], [106], [107]. Animal studies have shown that doses up to 16 mg are safe, and retinal and RPE changes begin to show when the dose is escalated to 20 mg [108], [109], [110], [111].

### 6.2 Structural changes

#### 6.2.1 Swelling of the arcuate nerve fiber layer (SANFL)

Transient increase in RNFL in the macular area is noticed as early as within 10 days of surgery and progressively reverts to baseline within 2 months. On infrared (IR) and autofluorescence (AF) photographs, this appears as dark thick stria arising from the edge of the optic nerve head (ONH) and extending towards the macula in an arcuate fashion. On scanning of the corresponding section by spectral domain OCT (SDOCT), swelling of the arcuate NFL is revealed. SANFL is not visible on clinical examination or fundus photographs. It has however been shown to have no effect on the visual outcome. Inadvertent injury to the inner retina during peeling leading to disruption of axoplasmic flow along the nerves is thought to be one of the causes, and damage to Müller cell endplates due to the act of ILMP another one [112].

#### 6.2.2 Dissociated optic nerve fiber layer (DONFL)

DONFL was first described by Tadayoni et al. [113] after ERM surgery as multiple arcuate striae running along the course of the nerve fiber in the macular area, which were relatively darker than the surrounding retina on blue filter photographs. It could also be seen on red free photographs and on fundus examination, albeit faintly. It was seen in 43% of Tadayoni et al.’s cases 3 months after ERM surgery [113]. Of the 23% of the ERM specimens sent for analysis, ILM was present in all of them, but DONFL was seen in only 35.7% of these [113]. Thereafter, there were others who reported the occurrence of DONFL following ILMP for idiopathic MH [114], [115]. On OCT, there were pittings or dimples in the inner retinal layer corresponding to the striae, which were not connected with each other. Mitamura et al. [116] found the depth of dimples to be less than the RNFL thickness, while in a study by Nukada, the dimples extended to variable depths from RNFL to IPL, with the deeper ones being more in the temporal macula, which may have been more evident because of thinning of the temporal macula. The number of dimples on the other hand was the least in the temporal region, followed by nasal, inferior, and superior in increasing order [117]. Alkabes et al. [118] described the appearance of DONFL on en face OCT as multiple dark spots along the course of NFL, terming them ‘concentric macular dark spots’ (CMDs). It is observed to occur between 1–3 months in the macular area denuded of ILM with no spontaneous resolution. Liu et al. [118] reported three patterns of distribution: dark striae running along the nerve fibers sparing temporal raphe; the second type is concentrating over the papillomacular bundle; and the final type is scattered over the peeled area, which was more commonly seen in myopic eyes. Progression in terms of increase in number and size was noted up to 6 months [119], [120]. There have been a few speculations regarding what it is and how it came to be. Tadayoni et al. [113] attributed the peculiar appearance to the dissociation or separation of optic nerve fibers, which may have been the result of mechanical traction exerted by peeling of ERM and ILM, and/or because of the injury to the Müller cells, which bundles up the nerve fibers together. Tadayoni et al. [113] also had an alternative explanation, according to which the appearance of DONFL could simply be the natural rough surface of optic nerve fibers with Müller cell processes which has now been exposed after ILMP [113]. However, the arguments against this are that the depth of the dimple is more than the thickness of ILM [116], extending to deeper levels, and a dimple is not seen in all the cases that undergo ILMP. Its incidence is noted to be 43–100% [113], [115], [118], [119], [121]. There are other schools of thought like the regeneration of Müller cells processes following the trauma [121], [122] or the gradual degeneration of Müller cell end feet [123] might be the probable causes of cleavage of nerve fibers leading to the typical appearance. Spaide [121] also thought the pattern of appearance could have been because of the non-uniformity in the distribution of Müller cells, like density is more between nerve fiber bundles; hence following avulsion of Müller cell endplates, the alterations are more evident in such areas. The void created by such avulsion is initially masked by RNFL swelling which shows itself after the swelling subsides [120], as is observed by Clark who deduced that SANFL and DONFL are two stages of the same pathological process initiated by ILMP [112]. Spaide observed localized thinning of the ganglion cell layer (GCL) in the areas with dimples, which was thought to be due to the loss of Müller cells or their processes, because loss of ganglion cells would mean attenuation of RNFL proximal to dimple and visual field defects [121], but DONFL is not associated with poor visual acuity or decreased retinal sensitivity or visual field defects [113], [115], [48], [124]. Focal macular electroretinogram post-ILMP has resulted in delayed and limited recovery of b-wave amplitude, suggestive of Müller cell injury [125]. The severity of DONFL has been found to be associated with the amount and the size of cellular debris on the retinal side of the peeled ILM [126]. The choice of instrument – forceps vs. diamond-dusted membrane scraper (DDMS) – used for the entire peel also determines its prevalence and the extent. DDMS, either by altering the plane of cleavage or by increasing the pressure applied on the retina, has been found to be responsible for higher frequency and severity of DONFL [127].

#### 6.2.3 Alteration of RNFL thickness

Peripapillary RNFL thickness is increased within a month and has been reported to last for varying periods of time from 3 to 12 months without any corresponding visual field defect. RNFL thickness in some sectors progressively decreased to below basal level, although there is no uniformity in the reports regarding the sector involved [128], [129], [130]. The factors thought to be associated with this occurrence are dye toxicity, intraocular fluids/gases used, and direct trauma to ONH during PVD induction. However, Toba et al. [130] in their study found that the type of dye did not play any role in the alteration of RNFL thickness.

#### 6.2.4 Attenuation of ganglion cell complex (GCC)

GCC comprises of nerve fiber layer (NFL), ganglion cell layer (GCL), and inner plexiform layer (IPL) containing axons, cell bodies, and dendrites of ganglion cells, respectively. Baba et al. [131] reported decrease in thickness of GCC, mostly temporal and inferior to the fovea, after 6 months of ICG-assisted ILMP, which was thought to be due to injury of GCC given its close proximity to ILM, and due to dye toxicity. Another study [132], however, concurred in the occurrence of thinning of GCC, and showed that it was not related to ICG. There was no significant difference in GCC thickness between the ICG and BBG group [132]. The attenuation of GCIPL over the temporal area has been supported by Sabater et al. [133] with the explanation that relatively thin RNFL over the temporal macula might have been unable to protect ganglion cells from toxic effects of BBG and from mechanical trauma inflicted by ILMP. SD-OCT-based analyses of cases 3–6 months after MH surgery with brilliant blue-assisted ILMP have demonstrated increase in retinal thickness medial to the fovea while thinning temporally [134], [135]. However, thinning of GCL occurred on either side, more so temporally [134]. Faria et al. [135] found thinning of RNFL, GCL, and IPL on either side of the fovea, but significantly more on the temporal side, which they thought to have been a result of local inflammation and stretching effects. On the contrary, Sevim et al. [136] stated that BBG-assisted ILMP caused no significant change in RNFL and GCC thickness.

#### 6.2.5 Shortening of papillofoveal distance

With the thinning of the temporal macula, thickening of the nasal part occurred with nasal displacement of the fovea after 6 months of the surgery [137], [138], [139], [140], [141]. These changes were also seen when ILMP was done for indication other than MH like diabetic macular edema (DME) [142]. Kawano et al. [138] found significant foveal migration towards disc after MH with TA-assisted ILMP, while it was not seen in the cases of spontaneous hole closure. There was no association of degree of displacement with the size of MH and the post-operative BCVA [138]. On the contrary, a correlation was seen between the ratio of migration of the temporal retina and the basal diameter of MH in a study by Ishida et al. [139]. Also, there was greater shifting of the temporal retina towards the optic disc than nasal [139]. Sun et al. [141] noticed that the position of the macula was not altered during the formation of macular hole, but after surgical closure with BBG-assisted ILMP, a new foveal pit was found nasal to the original fovea. ILM is basal lamina of the Müller cells. Histological evaluation of peeled ILM has shown small fragments of Müller cell end feet attached to its undulating part [143]. In the absence of the structural support provided by intact Müller cells, mobility of neurosensory retina (NSR) might increase. Other hypotheses are that ILMP by some unknown cause might lead to depolymerization of microtubules, thus causing axons to shrink, or some intravitreal chemical factors interacting with exposed nerve fibers in the peeled area might cause them to shrink. The nerve fibers being anchored to the lamina cribrosa are pulled towards the disc after the shrinkage [139]. Another explanation is that NFL follows a zigzag course following ILMP, which might cause contractile forces to act towards the optic disc. Shortening of the papillofoveal distance probably leads to stretching and thinning of the temporal retina, or the other way round might be the case. Thinning of the temporal retina might be the result of atrophy or degeneration causing the biomechanical forces to tip off to the nasal side with resultant nasal foveal migration [142].

### 6.3 Functional changes

There have been conflicting reports of this aspect of ILM peeling. Haritoglou et al. [144] reported paracentral scotoma in 56.2% of cases after ILMP. They were relative (27.1%) and deep (72.9%), single or multiple, but did not interfere with good visual acuity. They were more in the temporal region, followed by superior and inferior, and the least in the nasal area [144]. This was confirmed by Tadayoni et al. [145], who – using OCT/SLO microperimetry – found reduced mean differential light threshold sensitivity by 3.4 dB in addition to absolute scotoma in patients who had undergone ILMP. The etiology could be forceps-related retinal trauma or secondary degeneration [11]. Ito et al. [115], on the other hand, found no scotoma by SLO microperimetry in the eyes with DONFL, nor was the BCVA and macular sensitivity measured by Humphrey 10-2 any different between the eyes with or without DONFL. Similarly, sensitivity in central 10° was observed by Christensen et al. [48] to be comparable among ICG-peeled, TB-peeled, and non-peeled groups. Runkle et al. [124] also found no difference in the sensitivity between eyes with or without DONFL, or between the nasal and temporal quadrant in eyes with DONFL, or between ILM-peeled eye and the fellow eye. There was no difference in sensitivity when the area across DONFL and normal retina around it was evaluated with MP-1 microperimetry [146]. And when it was assessed with MP-3 in the area around the MH after ILMP, the retinal sensitivity was unexpectedly found to be increased at both 1 and 4 months postoperatively in 84.1% of the cases. The cases in which the sensitivity was decreased belonged to the older age group, which was thought to be associated with retinal recovery ability [147]. Besides achieving anatomical success and improvement in BCVA, Cappello et al. [148] also found an increase in the maximum reading speed and retinal sensitivity post-ILMP for MH.

## 7 Alternatives

The anatomical outcome of MH surgery has been excellent with ILMP, but the reports of its effects on the structure and function of the retina have been a topic of debate. This has encouraged surgeons to continue their quest for finer techniques and alternative treatment options.

### 7.1 Temporal ILM flap/hemi-temporal ILM peeling

Michalewska et al. [25] while introducing inverted ILM flap had shown it to be superior to conventional vitrectomy with ILM peeling in terms of closure rate (98% vs. 88%) and rate of flat/open type of closure (0%). With increasing reports of side effects related to ILMP, the need of a new technique was felt which would give an equally good outcome, but with a lower risk of side effects. Michalewska et al. [25] then modified their own technique such that ILM is peeled only in the temporal quadrant over an area of 2DD up to the edge of MH, and is then reflected onto the MH. This technique gave an equally good anatomical and visual outcome as the inverted flap technique and with DONFL limited to the temporal region [149]. Being a safe and effective technique, its indication has broadened to include large, chronic, myopic MH, those who cannot maintain prone position [150], [151], and traumatic MH [152]. However, the cases of MH with ERM tend to result in post-operative distortion of the fovea due to recurrence of ERM in the region where ILM is not peeled [150].

Shiono et al. [153] described peeling of ILM over the region temporal to MH without the formation of the flap in idiopathic MH of <1000 µ diameter. The primary closure rate was comparable to the 360° ILM peeling group (93.3% vs. 92.5%), and so was the post-operative gain in visual acuity. The displacement of the temporal retina was more than nasal retina in both the groups, as previously reported by Ishida et al. [139]. Migration of nasal retina towards the optic disc was significantly less in the hemi group after one week, but the difference ceased to remain statistically significant after one month of surgery. The shift of temporal retina towards the optic disc was similar in both groups. The effects beyond one month post-operative period is not known [153].

### 7.2 ILM abrasion

A novel technique of MH surgery by ILM abrasion in 1DD area around the MH using diamond-dusted membrane scraper (DDMS) with promising results of a 94% closure rate has been introduced. Using the more rounded surface, the scraper is moved over the macular surface – first in a circumferential manner around the hole at the distance of 1DD, and then in a radial manner from the same distance toward the hole in multiple strokes all around the hole. The study included MH of stages 2, 3, and 4, and there was no significant difference in closure rates between the first and the latter two stages. None of the closed MH reopened during 3–12 months of the follow-up period. Visual acuity of ≥20/40 was attained in 35% and that of ≥20/50 in 52% three months after surgery [154]. This technique, when done with the right amount of pressure, removes only cellular membrane and the surface layer of ILM, which has been found to be enough to initiate the reparative process. This avoids damage of other sensitive inner layers. However, if the pressure is less, ERM is not completely removed, which may lead to failure of closure of the hole. On the other hand, if the pressure is more, it leads to disruption of ILM and possible damage of NFL [155].

### 7.3 Ocriplasmin (OCP)

Ocriplasmin is a truncated human plasmin when injected at the dose of 125 µg/0.1 ml lyses laminin and fibronectin, two of the components of vitreo-retinal interface causing posterior vitreous detachment. RCT have shown that it causes closure of MH of less than 400 µ diameter associated with vitreomacular adhesion (VMA) in 30–40.6% [156], [157]. The success is greater in eyes with MH ≤250 µ diameter (47.8–58.3%) in comparison to MH of >250–400 µ diameter (23.5–36.8%) [156], [158]. With the anatomical success, improvement in visual function was also obtained as indicated by BCVA and the 25-item visual function questionnaire (VFQ-25). BCVA at 6 months improved by ≥2 lines in 72.1%, and by ≥3 lines in 48.8% of the FTMH cases which had achieved closure by day 28, together with better scores in VFQ-25 assessment in comparison to the ones which had not achieved closure by the given time [159]. Reopening of MH occurred in 9.3% [160]. PPV for persistent VMT/MH following administration of OCP had a similar outcome as PPV-only [161], [162]. With experience it has been known that OCP works best when certain criteria are met, such as age ≤65 years, phakic lens status, FTMH <250 µ, VMA <1500 µ, absence of ERM, macular pucker, diabetic retinopathy, and history of previous intraocular surgery [163].

Regarding its safety profile, MIVI TRUST and OASIS have reported adverse effects to be mild and transient. Floaters was the most common adverse event, followed by photopsia [156], [157]. Hahn et al. [164], in a study analyzing pre-marketing and post-marketing adverse effects of OCP, divided it into eight groups: acute reduction in vision secondary to progression of VMT and/or MH or to occurrence of sub retinal fluid, electroretinogram (ERG) changes, dyschromatopsia, retinal tears and detachments, lens subluxation and phacodonesis, impaired pupillary reflex, ellipsoid zone findings, and retinal vessel findings. Nyctalopia with reduction of a and b wave amplitudes on scotopic ERG has been reported [165]. The panretinal abnormalities are thought to be due to the action of OCP on laminin present throughout the retina and in various layers namely the Bruch’s membrane, the interphotoreceptor matrix, the external limiting membrane, the outer plexiform layer, the inner plexiform layer, and the internal limiting membrane [166].

## 8 Conclusion

ILMP since its introduction has been embraced by VR surgeons for the surgeries related to VRI including MH. Adverse effects related to it, however, have been of concern, and as a result have led to several modifications and search for alternatives. Temporal ILM flap has emerged as an equally effective procedure with the added benefit of a limited area of structural changes. Future prospective randomized studies with long-term follow-up can compare the newer techniques with ILMP in terms of anatomical and functional outcome. Vitrectomy alone also has shown successful anatomical results in MH <400 µ. If the risk related to vitrectomy is also to be avoided, OCP can be an option especially for MH <250 µ, but with a lower success rate and its own list of side effects. The risks, benefits, and cost effectiveness of each procedure should be weighed and discussed with the patient before deciding on the mode of treatment.

## Notes

### Authors’ ORCIDs

- Deepti Pradhan: 0000-0002-2595-181X
- Lalit Agarwal: 0000-0002-2681-768X
- Ichhya Joshi: 0000-0002-1813-6008
- Anamika Kushwaha: 0000-0002-6953-8250
- Kshitij Aditya: 0000-0002-9276-7756
- Archana Kumari: 0000-0001-7930-9446

### Acknowledgment

The authors are grateful to Mr. Shailendra Jha and Ms. Theresa Weippert for helping with the translation of the title and the abstract into German.

### Competing interests

The authors declare that they have no competing interests.
